# Role of gamma-delta T cells in host response against *Staphylococcus aureus*-induced pneumonia

**DOI:** 10.1186/1471-2172-13-38

**Published:** 2012-07-09

**Authors:** Ping Cheng, Tao Liu, Wei-Ying Zhou, Yuan Zhuang, Liu-sheng Peng, Jin-yu Zhang, Zhi-Nan Yin, Xu-hu Mao, Gang Guo, Yun Shi, Quan-ming Zou

**Affiliations:** 1 Department of Clinical Microbiology and Immunology, Faculty of Medical Laboratory Science, Third Military Medical University and National Engineering Technological Research Center of Immunological Biologicals, Chongqing 400038, China; 2 Department of Pharmacology, College of Pharmacy, The Third Military Medical University, Chongqing, China; 3 College of Life Sciences, Nankai University, Tianjin, China

## Abstract

**Background:**

*Staphylococcus aureus* is the major cause of hospital-acquired and community-acquired pneumonia. Host defense to *S.aureus* infection is largely mediated by the innate immune system. γδ T cells play an important role in innate immunity to many infectious diseases. However, less is known about the role of these cells during *S.aureus*-induced pneumonia. In this study, we examined the response and the role of γδ T cells to pulmonary *S.aureus* infection.

**Results:**

Mice infected with *S. aureus* intranasally showed rapid γδ T cells accumulation in the lung. Deficiency of γδ T cells led to attenuated bacterial clearance and less tissue damage in lung compared with WT mice. Moreover, TCR-δ^−/−^ mice exhibited impaired neutrophil recruitment and reduced cytokine production at the site of infection. The γδ T cells in response to pulmonary *S. aureus* infection mainly secreted IL-17 and γδ T cells deficiency reduced IL-17 production, which might regulate the production of neutrophil-inducing cytokine/chemokine in the *S. aureus*-infected lungs.

**Conclusions:**

Accumulation of γδ T cells in the lungs to *S. aureus* infection is beneficial for bacteria clearance and also contributes to the tissue damage. These cells were the primary source of IL-17, which might influence the recruitment of neutrophils at the early stage of infection.

## Background

*Staphylococcus aureus (S.aureus)* is a Gram-positive, extracellular bacterium that cause a variety of infections, including pneumonia, septic arthritis, bacteremia, endocarditic and cellulitis [1]. *S.aureus*-induced pneumonia accounts for 20-50% nosocomial pneumonia and 25.5% of community-acquired pneumonia, which causes especially severe pulmonary infection and is associated with high morbidity and mortality [2,3]. In recent years, the rapid emergence of Methicillin-resistant *S.aureus* raises great concern about the cellular and molecular mechanisms of host defense against *S.aureus* infection.

Innate immune response plays a critical role in the host defense against *S.aureus* infection. Rapid and massive neutrophil recruitment to the site of infection is essential for host defense against invading pathogenic microbes [4]. In a mouse model of *S. aureus* pneumonia, depletion of neutrophils resulted in delayed bacteria clearance and decreased survival of infected mice [5]. Following *S.aureus* challenge, alveolar macrophages and dendritic cells (DC) are also recruited to the site of infection and contribute to bacterial clearance, but might also exacerbate lung inflammation and injury [6,7]. Thus, early interactions between these innate cells and pathogens are important in determining the outcome of *S.aureus*-induced pneumonia.

In addition to these conventional innate cells, T cells bearing the γδ TCR (γδ T cells), another subset of innate immune lymphocytes, play an essential role in the mucosal host defense bacteria infection. Previous studies suggest that γδ T cells play an important role in regulating the initial immune response to lung pathogens via influencing the recruitment of neutrophils [8], DC or macrophages [9]. Although the lung γδ T cells comprise a very small percentage of pulmonary immune cells [10], they seem to be the first line of immune response against invading microbial pathogens. Depletion of γδ T cells led to impaired host defense to lung infections by *Klebsiella pneumonia*[11], *Mycobacterium tuberculosis*[12] or *Streptococcus pneumoniae*[13]. It has been reported that γδ T cells also play an important role in cutaneous *S. aureus* infection [14]. However, the exact roles of these cells played in *S.aureus*-induced pneumonia have not been well defined.

In the present study, we first observed an accumulation of γδ T cells in lungs infected with *S. aureus*. To investigate their role during an acute *S. aureus* pneumonia, we assessed the effects of the deficiency of γδ T cells on the host defense against *S.aureus* infection. Our results demonstrate that γδ T cell knock out attenuated bacterial clearance and resulted in less severe lung damage. Furthermore, a defect of IL-17 induction and impaired neutrophil recruitment following *S. aureus* challenge was observed in the absence of γδ T cells. We presumed that the production of IL-17 by γδ T cells had effects on early neutrophil-mediated lung inflammation and acute tissue injury.

## Results

### γδ T cells were accumulated in the lungs infected with *S. aureus*

**Figure 1:**
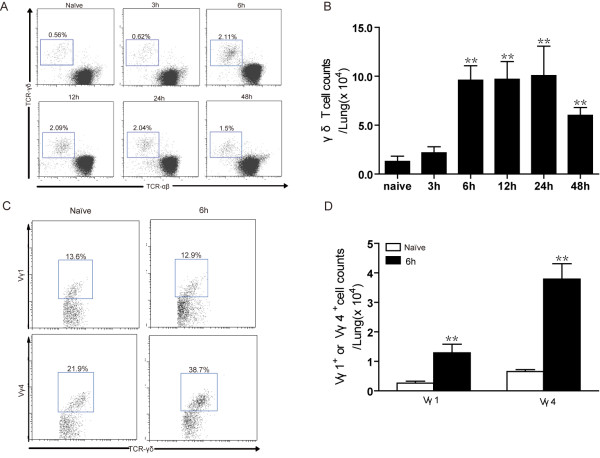
**Pulmonary γδ T cells are increased following *S. aureus *infection.** C57BL/6 mice were intranasally infected with SA 75 strain or PBS as mocked infection and sacrificed at different time post infection. (**A**) Pulmonary leukocytes were isolated and TCRαβ^–^ TCRγδ^+^ cells were detected by flow cytometry. Cells were gated on CD3^+^ cells. Representative flow cytometry dot-plots were shown. Numbers represent the percentage of γδ T cells. (**B**) Absolute numbers of γ δ T cells in the total lung at various time points following *S.aureus* challenge were shown. (**C**) The expression of Vγ1 and Vγ4 chains on gated CD3^+^ TCRαβ^-^ TCRγδ^+^ lung cells were analyzed by flow cytometry. Numbers indicate percentages of Vγ1^+^ or Vγ4^+^ γδ T cells. Representative flow cytometry dot-plots were shown. (**D**) Absolute numbers of Vγ1^+^ or Vγ4^+^ γδ T cells in naive and 6 h post-infection lungs. Bars represent mean ± SEM of 3–6 mice/time from three independent experiments. ** *p* < 0.01 versus naive control.

To investigate whether γδ T cells are involved in host response to *S. aureus* infection, the kinetics of the γδ T cell response in the lungs of mice infected with *S.aureus* was examined by flow cytometry. In mock-infected mice, γδ T cells comprised only about 0.5% of the lung CD3^+^ T lymphocytes, whereas the percentage was significantly increased from 6 h post-infection (Figure 1A). While similar kinetics was observed in the absolute numbers of γδ T cells in the total lung following *S. aureus* challenge (Figure 1B). Vγ1^+^ and Vγ4^+^ subsets are the two major subsets of lung γδ T cells. To determine which subsets of pulmonary γ δ T cells accumulated at the infected sites, we examined the proportion of Vγ1^+^ and Vγ4^+^ γ δ T cells in the lungs at 6 h post-infection. As shown in Figure 1C, Vγ1^+^ cells (identified as CD3^+^ TCRαβ^-^ TCRγδ^+^ Vγ1^+^ cells) were about 13.1 ± 3.1% (n = 6) of total γδ^+^ T cells in mock-infected mice, and the proportion of Vγ1^+^ cells was maintained following bacteria challenge. Nevertheless, considering the dramatically increased number of lung cells in response to acute bacterial infection, absolute numbers of Vγ1^+^ cell was significant increased at 6 h post-infection with *S.aureus* (*p* < 0.01 Figure 1D). Vγ4^+^ γδ T cells (identified as CD3^+^ TCRαβ^-^ TCRγδ^+^ Vγ4^+^ cells) comprised approximately 21.7 ±2.2% (n = 6) of γδ^+^ T cells in the mock-infected lung and were significantly increased in response to *S. aureus* challenge (mean 37.9 ± 5.2%). Consistently, their absolute number almost increased up to 6-fold at 6 h post infection (Figure 1D). Thus, these results demonstrated that pulmonary *S. aureus* infection results in an accumulation of γδ T-cell in the lung.

### Deficiency of γδ T cells led to attenuated bacterial clearance but less acute severe lung damage

**Figure 2:**
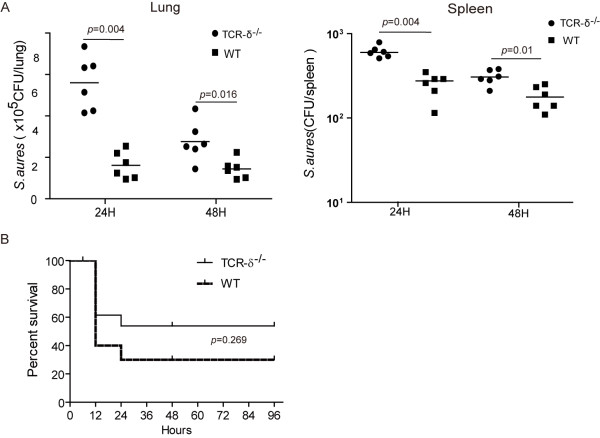
**Deficiency of γδ T cells leads to increased bacteria burden.** (**A**) Mice were inoculated with sub-lethal SA 75 strain (5x10^8^CFU,n = 6/group/time point). Bacterial burden in lung and spleen were determined at 24 and 48 hours following pulmonary infection with *S.aureus*. Each dot represents the bacterial count of the respective organ of a single animal. The line represents the median of each group. (**B**) Survival curve of WT(n = 8)and TCR-δ^−/−^ (n = 9) mice after intranasally inoculated with 5x10^9^ CFU bacteria. *p* = 0.269 for infected TCR-δ^−/−^ versus WT mice.

**Figure 3:**
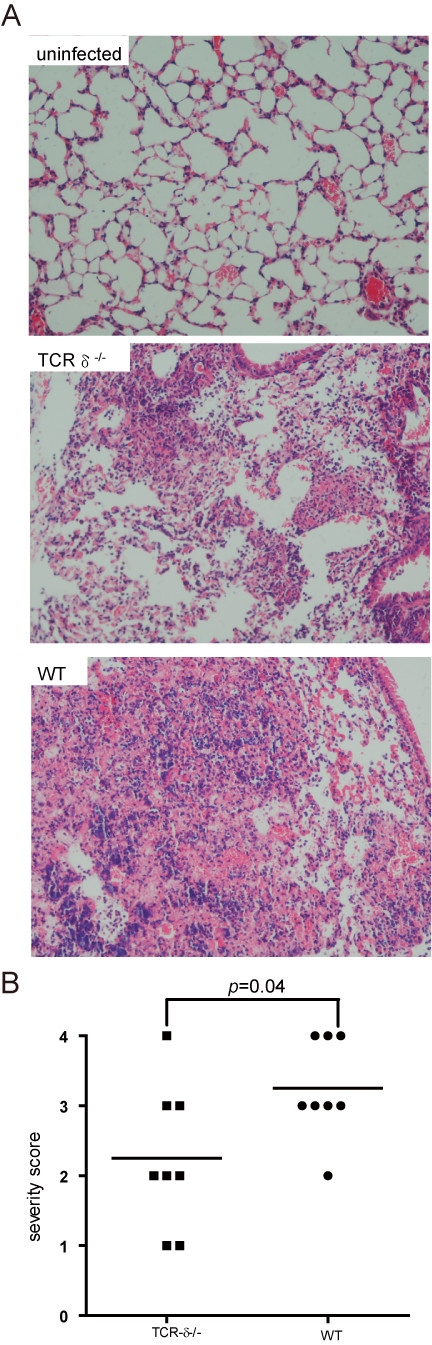
**The absence of γδ T cells leads to less severe lung lesions after *S. aureus *-induced pneumonia infection.** Lungs were harvested 12 hours following *S. aureus* challenge. (**A**) Representative H-E staining of lung sections from mock-infected control mice, TCR-δ^−/−^ mice and WT mice and (**B**) Histological scores of pneumonia in WT and TCR-δ^−/−^ mice (n = 8/group) were shown.

In order to investigate the role of γδ T cells in *S. aureus*-induced pneumonia, we next examined the effect of deficiency of γδ T cells on the host response to *S. aureus* infection. Initially, we compared the bacterial clearance and morbidity of TCR-δ^−/−^ mice and wild-type (WT) following an intra-nasal instillation of sub-lethal *S.aureus* infection (5x10^8^ CFU). The bacterial burden in lungs and spleens at both 24 and 48 hours post-infection was significantly higher in TCR-δ^−/−^ mice than that in WT mice (Figure 2A). Next, the survival rate of TCR-δ^−/−^ mice and WT mice after challenge with a lethal dose of *S.aureus* (5x10^9^ CFU) was analyzed (Figure 2B). Both WT and TCR-δ^−/−^ mice started to die at 12 hours and no significantly different mortality rate was observed. However, there were significant differences in pulmonary histology between TCR-δ^−/−^ mice and WT mice. As a control, uninfected WT mice showed normal lung parenchyma. At 12 hours post-infection, the prominent destruction of alveolae and the infiltration of large amounts of inflammatory cells were observed in WT mice, whereas less immune cell infiltration was seen in TCR-δ^−/−^ mice (Figure 3A). Overall the severity of pneumonia in TCR-δ^−/−^ mice was significantly lower than that in WT mice (Figure 3B). These results demonstrate that the lack of γδ T cells led to attenuated bacterial clearance but less severe acute lung damage.

### Lung neutrophils infiltration in response to *S. aureus* pulmonary infection was impaired in TCR-δ^−/−^ mice

**Figure 4:**
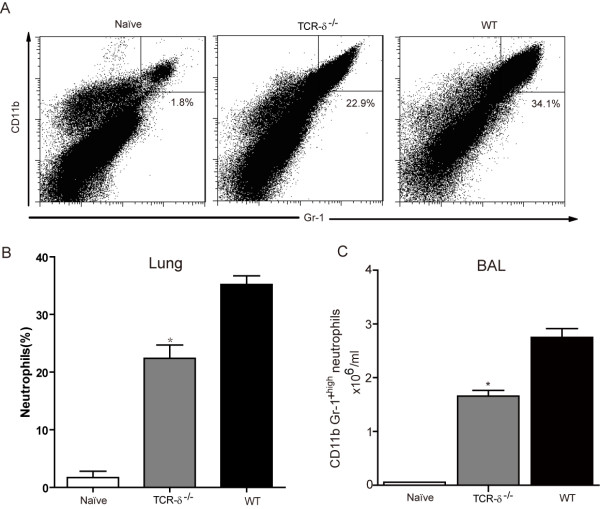
**γδ T cell–deficient mice have markedly impaired neutrophil recruitment during *S. aureus *-induced pneumonia.** WT and TCR-δ^−/−^ mice (3–6 mice/group) were intranasally inoculated with *S.aureus*. Cells were prepared from the lung at 6 h post infection and neutrophils were detected by flow cytometry. The representative dot-plots (**A**) and statistical results (**B**) were shown (n = 6). (**C**) Lungs of TCR-δ^−/−^ mice and WT were lavaged and the number of neutrophils in BAL fluid was determined by flow cytometry. *p < 0.05 versus WT.

Neutrophils provide an essential defense against invading *S. aureus*[4]. To test whether neutrophils are involved in the attenuated bacterial clearance in TCR-δ^−/−^ mice during *S.aureus* pneumonia, the neutrophils in the lungs at 6 h post-challenge were examined by flow cytometry. Neutrophils were identified by co-expression of CD11b and Gr-1. Compared with uninfected mice, infected WT mice exhibited a significant increase of CD11b^+^ Gr-1^high^ cells, whereas, neutrophils in infected TCR-δ^−/−^ mice were significantly lower than that in infected WT mice (Figure 4A&B). Furthermore, the number of neutrophils (CD11b^+^ Gr-1^high^) in BAL fluid of infected mice was examined. Fewer neutrophils were detected in the BAL fluid of TCR-δ^−/−^ mice as compared to wild-type mice (Figure 4C). These results suggest that the deletion of γδ T cell impair neutrophil recruitment to the lungs after infection with *S.aureus*.

### IL-17-producing γδ T cells provided early responses to *S. aureus* challenge

**Figure 5:**
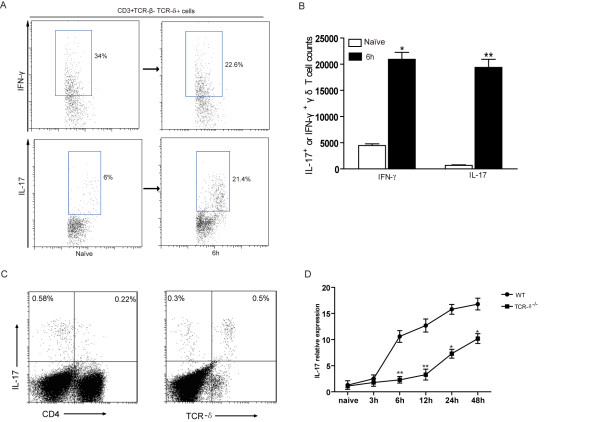
**Increase of IL-17-producing γδ cells following *S. aureus *challenge and reduced IL-17 induction in γδ T cells deficient mice.** Total lung cells were isolated from naive or *S.aureus*-challenged mice at 6 h post-infection and IL-17 and IFN-γ producing γδ^+^ T cell were detected by intracellular staining. Cells were gated on CD3^+^ TCRαβ^-^ TCRγδ^+^ cells. (**A**) The representative dot-plots were shown. Numbers indicate the percentages of IFN-γ^+^ cells (up panels) and IL-17^+^ cells (lower panels) among γδ T cells. (**B**) Absolute numbers of IFN-γ^+^ or IL-17^+^ γδ T cells in naive and 6 h post-infection lungs. (**C**) The representative dot plots showed the percentages of IL-17 producing cells in lung CD3^+^ T lymphocytes. Numbers in the quadrant indicate the percentage of cells. (**D**) IL-17A mRNA expression in the lungs following *S. aureus* challenge was determined by quantitative real-time PCR. Data are expressed as the mean ± SEM of 3–6 mice/time from three independent experiments. *p < 0.05 or **p < 0.01 versus WT mice.

IL-17 is an important proinflammatory cytokine involved in the migration and activation of neutrophils, and it is mainly produced by γ δ T cell in several infectious diseases [14,15]. To examine whether γδ T cells produce IL-17 after *S.aureu*s infection, production of IFN-γ and IL-17 by γδ T cells was analyzed by intracellular staining. We observed that the percentage of IFN-γ^+^ cells among γδ T cells was slightly decreased in lungs at 6 h post-infection. Whereas, accounting for the increased total γδ T cells number, there was a significant increase of absolute number of IFN-γ^+^ γδ T cells following *S.aureus* challenge (Figure 5B). Meanwhile, a significant increased proportion of IL-17^+^ γδ T cells were also observed. Consistently, their absolute number almost increased up to 30-fold at 6 h post infection (Figure 5A&5B). Further analysis by flow cytometry showed that γδ T cells are the primary source for IL-17 in the lungs of *S.aureus* infected mice. About 60% of lung IL-17-producing T cells was TCR-δ^+^, and few CD4^+^ T cells expressed IL-17(Figure 5C). We next analyzed if γδ T cell-deficient mice show any changes on the level of IL-17 after *S.aureus* infection. WT mice displayed an early burst of IL-17 expression, which increased at 6 hours and was sustained at a high level at all subsequent time points evaluated. In contrast, a significantly lower expression of IL-17 mRNA in the lungs of γδ T cell–deficient infected mice was observed from 6 hours post-infection onwards. In addition, γδ T cell–deficient mice exhibited delayed onset of IL-17 induction in infected lungs (Figure 5C). Together, these data implied that lung IL-17-producing γδ T cell provide early responses to bacteria challenge and deficiency of γδ T cells results in a defect of IL-17 induction after *S.aureus* infection.

### Decreased expression of neutrophil-inducing cytokine/chemokine in infected lungs of TCR-δ^−/−^ mice

**Figure 6:**
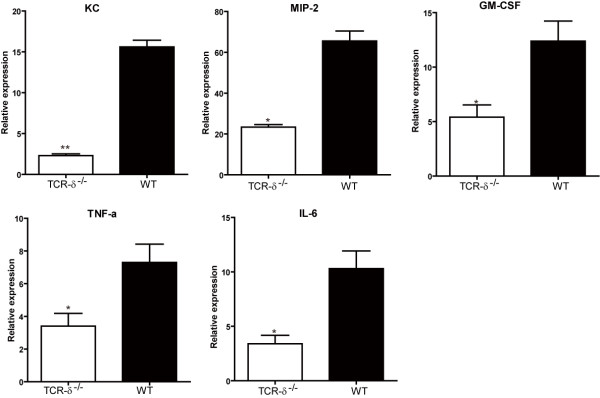
**Decreased expression of neutrophil-inducing cytokine/chemokine in the absence of γδ T cells.** The mRNA expression of neutrophil-inducing cytokine/chemokine from the lungs was performed by quantitative real-time PCR 6 hours after *S. aureus* inoculation. Data are expressed as the mean ± SEM of 3–6 mice/time from three independent experiments. *p < 0.05 or **p < 0.01 versus WT mice.

IL-17 regulates the production of CXC chemokines such as murine MIP-2/CXCL2, KC/CXCL1, which are necessary for neutrophil recruitment. Also, IL-17 participates in the induction of cytokines required for neutrophil differentiation and activation, including GM-CSF, IL-6 and TNF-α [16]. To further elucidate the relationship between the impaired neutrophil recruitment and decreased IL-17 production in TCR-δ^−/−^ mice, we compared the expression of these neutrophil-inducing cytokines/chemokines in the lungs of *S.aureus* infected TCR-δ^−/−^ and WT mice at 6 h post-infection. As shown in Figure 6, KC and MIP-2 production were severely impaired in the lungs of infected TCR-δ^−/−^ mice in comparison to WT mice. Similarly, the expression of GM-CSF, IL-6, and TNF-α were also significantly reduced in the TCR-δ^−/−^ mice, compared with those in the wild-type mice. These results implied that the impaired neutrophil recruitment in the absence of γδ T cell was caused, to some extent, by the decreased expression of neutrophil-inducing cytokine/chemokine at the early stage of infection.

## Discussion

In this study, we investigated the role of γδ T cells in the early host response to *S. aureus* infection. A significant accumulation of γδ T cells at lungs was observed as early as 6 h post-infection. The local expansion of resident γδ T cells and/or recruitment from the peripheral circulation may be responsible for the accumulation of γδ T cells in the lungs following *S.aureus* infection. In murine lungs, Vγ1^+^ and Vγ4^+^ γδ T cells subsets are two major subsets [10,13]. Generally, Vγ4^+^ γδ T cells are the dominant population of lung γδ T cells and Vγ1^+^ T cells are mainly located in lymphoid organs. Both subsets are involved in the immune response to various pulmonary infectious pathogens. For example, in the lungs of BCG-infected mice, substantial Vγ1^+^ and Vγ4^+^ γδ T cells accumulate at the site of infection. Concomitant with the expansion of pulmonary Vγ4^+^ γδ T cells, large amounts of Vγ1^+^ γδ T cells were recruited to the lungs of infected mice [17]. Also, both subsets of γδ T cells were significantly increased following *S. pneumoniae* challenge [13]. But conversely, Vγ4^+^ γδ T cells contributed to the increased size of γδ T cells and ablation of Vγ4^+^ γδ T cells attenuated neutrophil recruitment and impaired host protection [8]. Similarly, in our present study, both Vγ1^+^ and Vγ4^+^ γδ T cells were increased in the lungs. Whereas Vγ4^+^ γδ T cells were the dominant population of the increased pulmonary γδ T cells at the early phase of *S.aureus* infection. Compared with lung, no significant increase of γδ T cells in the spleen and draining lymph node of infected mice was observed (data not shown). We presume that accumulated γδ T cells were restricted to the lung at the early phase of *S.aureu*s infection. Of note, approximately 47% of γδ T cells expressing other Vγ genes were unidentified due to limited availability of antibodies in our lab. These cells may include remaining pulmonary Vγ2^+^ γδ T cells and/or Vγ6^+^ γδ T cells, as suggested by previous studies [17]. These subsets have not been well defined in the course of pulmonary infection. To determine whether γδ T-cell subsets have a differential role in the immune response to *S.aureus*-induced pneumonia will require further studies.

γδ T cells exert different influences on the host response in a variety of infection models. In some experimental animal models of pulmonary infections, γδ T cells were demonstrated to be protective against pathogens infection, such as *M. tuberculosis*[12], *Klebsiella pneumoniae*[8,11], *Nocardia asteroides*[18] and *Cryptococcus neoformans*[19]. In these infections the absence of γδ T cells rendered mice more susceptible to pathogen infection. In contrast, other studies reported that γδ T cells were not necessary to the host defense against microbial infection and even harmful to the host. Following challenge with *Candida albicans*[20] or *Salmonella choleraesuis*[21], γδ T cell knock out mice were resistant to infection and showed an accelerated pathogens clearance. So these data indicate that γδ T cell might exert diverse function in different infection disease depending on the unique of bacterial infection. In our current study, compared with wild-type mice, γδ T cell deficient mice show no significant change in survival. But the absence of these cells led to attenuated bacterial clearance. It appears that accumulated γδ T cells in the lungs might contribute to the host protective responses against *S.aureus* infection.

Contrastingly, regardless of bacteria load, we found that mutant mice exhibited less severe pulmonary lesions at the early stage of infection. Since neutrophils are responsible for the acute inflammation response to most infection in the lung, we next detected the neutrophils infiltration. The results showed that the infiltration of neutrophils in the lungs was less in TCR δ^−/−^ mice. It is well known that recruited neutrophils provide the first line of defense against many infections by ingesting the foreign invaders and limiting the pathogen survival and dissemination [4]. It is reported that depletion of neutrophils led to a fatal defect in murine pulmonary *S.aureus* clearance [5]. On the contrary, neutrophils have also been implicated in the pathology of many inflammatory conditions by releasing oxidants and hydrolytic enzymes [22]. Earlier studies reported that respiratory syncytial virus (RSV) could enhance neutrophils infiltration to the lung and the activated neutrophils significantly augmented RSV induced damage [23]. And pulmonary neutrophil infiltration is an insidious killer in polymicrobial sepsis, in patients with an acute bacterial infection, primed neutrophils with increased oxidative product formation contributed to the damage of pulmonary vascular endothelium during bacteremia [24]. Previous studies also reported that neutrophil recruitment during *S. aureus* USA300-induced acute pneumonia led to serious lung inflammation and injury, which was associated with the overwhelming release of cytotoxic granule contents [25]. Similarly, other reports demonstrated that the accumulated neutrophils at the site of infection may exacerbate the severity of *S. aureus* wound infection [26]. Our present study also suggested that the reduced infiltration of neutrophils might be responsible for both the increased bacterial dissemination and the less tissue damage in TCR δ^−/−^ mice.

Consistent with the decreased recruitment of neutrophils to *S.aureus* infection in the TCR δ^−/−^ mice, we also found decreased KC, MIP-2, GM-CSF, TNF-a, IL-6 and IL-17 in lung. These factors are known to regulate the neutrophil infiltration and their reduction might contribute to the less neutrophil infiltration. IL-17 is worthy of note among these factors, since it has been shown to be a crucial regulator of the migration and activation of neutrophils and also secreted by the γδ T cells. γδ T cells are responsible for much of this rapid cytokine production in the earliest stages of an inflammatory response [27]. Some groups reported that IL-17A-producing cells were essential for vaccine efficacy against systemic *S. aureus* infection [28,29]. Previous study reported that IL-17 deficient mice displayed impaired host defense against mucoepithelial infection by *S. aureus*[30]. Similarly, Kudva et al. described the impact of IL-17-mediated immune response against *S. aureus* pneumonia. Mice deficient in IL-17R showed impaired bacteria clearance from the lung [31]. In this study, we found that the main source of IL-17 in *S.aureus*-infected lung was γδ T cells and depletion of γδ T cells results in the reduced IL-17 expression in the early phase of infection. These results implied that the impaired neutrophil accumulation in γδ T cell-deficient mice was likely caused, at least in part, by the decreased production of IL-17 at the site of infection. Nevertheless, no direct evidence of the involvement of IL-17-producing γδ T cells was presented. We cannot exclude the possibility that other products of γδ T cells may have effects on the early neutrophil recruitment. The role for IL-17-producing γδ T cells in the *S.aureus*-induced pneumonia awaits further study.

## Conclusions

In conclusion, γδ T cells accumulated in the lungs after *S.aureus* infection, which was the primary source of IL-17 at the early phase of infection. Its presence is beneficial for bacteria clearance but also contributes the early acute inflammation and tissue injury in lung. The function of γδ T cells in *S. aureus*-induced pneumonia might be mediated by neutrophil infiltration. Overall, our findings provide insight into the complex innate immune system in early pulmonary *S.aureus* infection. Further study is required to elucidate the detailed mechanisms by which γδ T cells control the bacteria clearance and facilitate the tissue damage, which might be helpful to find a way to regulate the γδ T cell to benefit bacteria clearance and avoid the tissue damage.

## Methods

### Mice

Six- to 8-wk-old female C57BL/6 mice were purchased from the Experimental Animal Center of the Third Military Medicine University. B6.129P2-Tcrd^tm1Mom^/J (B6 TCR-δ^−/−^) mice were kindly provided by Dr. Zhinan Yin (College of Life Sciences, Nankai University, Tianjin, China). All mice were housed in specific pathogen-free facilities. All experiments were approved by the Animal Ethical and Experimental Committee of Third Military Medical University.

### Bacteria strains and mouse pneumonia model

The mouse model of *S. aureus* pneumonia was established as previously described [7]. Briefly, a clinical isolate of the *S. aureus* SA75 strain was cultured in Mueller Hinton Agar (MHA) for 24 hours prior to inoculation into Mueller Hinton broth which was then incubated overnight. Then the overnight culture was diluted 1:100 into fresh Mueller Hinton broth (MHB). Bacteria were grown at 200 rpm at 37°C to an optical density at 660 nm of 0.5. Cells were collected and washed twice with PBS. All mice were anesthetized with isoflurane and inoculated intranasally with 5 × 10^8^ or 5 × 10^9^ CFU of *S. aureus* SA75. Animals were held upright for 1 min post-inoculation. Mock-infected control mice were inoculated intranasally with sterile PBS. Mice were either sacrificed at pre-determined time points or assessed for survival for 48 h.

### Isolation of pulmonary leukocytes

Pulmonary leukocytes were isolated as previously described [19]. Briefly, the whole lungs were removed aseptically and cut into small pieces and incubated in RPMI 1640 (Hyclone) containing 20U/ml collagenase type I and 1 μg/ml DNase I (Sigma-Aldrich)for 1 h at 37°C under continuous rotation. And the lung portions were crushed through 40 µm meshed steel sieves. The total cell pellet was resuspended in 3 ml of 40% (v/v) Percoll (Pharmacia, Uppsala, Sweden) and layered onto 3 ml of 80% (v/v) Percoll. After centrifugation at 600x g for 20 min at room temperature, the cells at the interface were collected and washed in PBS. All isolated cells were enumerated and resuspended in RPMI 1640. Total viable cell counts were determined by trypan blue exclusion.

### Bronchoalveolar lavage (BAL)

Bronchoalveolar lavage (BAL) was performed as previously described [8]. Briefly, the chest was opened and the trachea was exposed through a midline incision and cannulated with a sterile 22-gauge Abbocath-T catheter and BAL was performed by instillation of two 0.5-ml aliquots of sterile saline. Approximately 0.8 ml of BAL fluid was retrieved per mouse.

### Bacterial counts

Mice were sacrificed at 24 h and 48 h postinfection and the levels of bacterial load were determined by preparing lung and spleen homogenates in PBS and plating serially dilutions on MHA. After 18 h of incubation at 37°C, colony forming units were calculated by standard plate counting and presented as CFU/organ. BAL was collected and plated 10-fold serial dilutions on MHA. Colonies were determined as CFU/ml.

### Histopathology

Lung tissue was fixed with paraformaldehyde and embedded in paraffin, sectioned, and stained with hematoxylin and eosin (H&E). A double-blind histological analysis was performed to examine the sections from each lung of mock-infected and infected mice, and each section was given a score of 0–4 (no abnormality to most severe pneumonia) according to established criteria [5].

### Flow cytometry

FACS was performed to analyze cell surface marker and intracellular cytokine expression. Briefly, Lung cells were isolated as mentioned above and stimulated for 6 h with PMA (50 ng/ml; Sigma-Aldrich, St. Louis, MO), ionomycin (1 μg/ml; Sigma-Aldrich) and Golgistop (BD Pharmingen, San Diego, CA). The cells were blocked with anti-CD16/32 antibody (clone 2.4 G2; BD Biosciences) on ice for 15 min, stained for 30 min with the following specific Abs: FITC-anti-γδ TCR(clone GL3), PECy5.5-anti-TCRβ (clone H57-597) and PerCP-Cy5.5-anti-CD8 (clone 53–6.7) were purchased from eBioscience(San Diego, CA), FITC-anti-GR-1(clone RB6-8 C5), APC-Cy7-anti-CD3 (clone 17A2), PerCP-Cy5.5-anti-CD4 (clone RM4-5) and PerCP-Cy5.5-anti-CD11b (clone M1/70) were from Biolegend, PE-anti-TCR Vγ1 (clone 2.11) and APC anti- TCR Vγ4 (clone UC3) were from Tianjin Sungene (Tianjin, China). Intracellular cytokine staining was performed after fixation and permeabilization, using Perm/Wash solution (BD Biosciences). The cells were then separately stained intracellularly with PE-Cy7-anti-IFN-γ (XMG1.2) and PE-anti-IL-17A (TC11-18 H10) (BD Biosciences). Samples were acquired on FACSCanto II (BD Biosciences). Data were analyzed with Flowjo software or FACSDiva software (BD Biosciences).

### Lung RNA isolation and Real-time PCR

Total lung RNA was isolated using Trizol (Gibco), according to the manufacturer’s instructions. DNA was removed from RNA preparations with DNase I digestion (Invitrogen) for 30 min at 37°C. And samples were reverse-transcribed to cDNA using the PrimeScript® RT Master Mix (Takara) according to the manufacturers’ instructions. Real-time PCR was performed on the iQ5 apparatus (BioRad, Hercules, USA). The cycle number at which the various transcripts were detectable, referred to as the threshold cycle (Ct), was compared with that of β2-M and referred to as ΔCt. The relative gene expression was expressed as fold change calculated by the ^ΔΔ^ Ct method. The expression of IL-17A was determined by the TaqMan method using primers and probes previously reported [32]. The mRNA of keratinocyte-derived chemokine (KC), macrophage inflammatory protein 2 (MIP-2), GM-CSF, tumor necrosis factor alpha (TNF-α) and IL-6 were measured by SYBR Green Realtime PCR Master Mix (Toyobo) using the previously described primers [15]. Mouse β2-M served as the normalizer.

### Statistical analysis

All data are expressed as means ± standard errors of the means (SEM). The statistical analysis software used was SPSS Version13.0. Normally distributed data were analyzed using Student’s *t*-test. Otherwise, the non-parametric Mann–Whitney test was used. Survival studies were analyzed using Kaplan-Meier testing. A *P* value of < 0.05 was considered to represent a statistically significant difference. The data were from at least three independent experiments.

## Competing interests

The authors declare that they have no competing interests.

## Authors’ contributions

Conceived and designed the experiments: QZ, YS and TL. Performed the experiments: PC, WZ, YZ, LP, and JZ. Analyzed the data: PC, TL, XM and YS. Contributed reagents/materials/analysis tools: PC, TL, WZ, ZY, XM and GG. Wrote the paper: PC, TL, YS, QZ. All authors read and approved the final manuscript.
